# Targeted disruption of glycogen synthase kinase-3β in cardiomyocytes attenuates cardiac parasympathetic dysfunction in type 1 diabetic Akita mice

**DOI:** 10.1371/journal.pone.0215213

**Published:** 2019-04-12

**Authors:** Yali Zhang, Charles M. Welzig, Marian Haburcak, Bo Wang, Mark Aronovitz, Robert M. Blanton, Ho-Jin Park, Thomas Force, Sami Noujaim, Jonas B. Galper

**Affiliations:** 1 Molecular Cardiology Research Institute, Tufts Medical Center, Boston, Massachusetts, United States of America; 2 Departments of Neurology and Physiology, Medical College of Wisconsin, Milwaukee, Wisconsin, United States of America; 3 Department of Medicine, Tufts University School of Medicine, Boston, Massachusetts, United States of America; 4 Division of Cardiovascular Medicine, Vanderbilt University Medical Center, Nashville, Tennessee, United States of America; 5 Molecular Pharmacology and Physiology, University of South Florida, Tampa, Florida, United States of America; University of Edinburgh, UNITED KINGDOM

## Abstract

Type 1 diabetic Akita mice develop severe cardiac parasympathetic dysfunction that we have previously demonstrated is due at least in part to an abnormality in the response of the end organ to parasympathetic stimulation. Specifically, we had shown that hypoinsulinemia in the diabetic heart results in attenuation of the G-protein coupled inward rectifying K channel (GIRK) which mediates the negative chronotropic response to parasympathetic stimulation due at least in part to decreased expression of the GIRK1 and GIRK4 subunits of the channel. We further demonstrated that the expression of GIRK1 and GIRK4 is under the control of the Sterol Regulatory element Binding Protein (SREBP-1), which is also decreased in response to hypoinsulinemia. Finally, given that hyperactivity of Glycogen Synthase Kinase (GSK)3β, had been demonstrated in the diabetic heart, we demonstrated that treatment of Akita mice with Li^+^, an inhibitor of GSK3β, increased parasympathetic responsiveness and SREBP-1 levels consistent with the conclusion that GSK3β might regulate IKACh via an effect on SREBP-1. However, inhibitor studies were complicated by lack of specificity for GSK3β. Here we generated an Akita mouse with cardiac specific inducible knockout of GSK3β. Using this mouse, we demonstrate that attenuation of GSK3β expression is associated with an increase in parasympathetic responsiveness measured as an increase in the heart rate response to atropine from 17.3 ± 3.5% (n = 8) prior to 41.2 ± 5.4% (n = 8, *P* = 0.017), an increase in the duration of carbamylcholine mediated bradycardia from 8.43 ± 1.60 min (n = 7) to 12.71 ± 2.26 min (n = 7, *P* = 0.028) and an increase in HRV as measured by an increase in the high frequency fraction from 40.78 ± 3.86% to 65.04 ± 5.64 (n = 10, *P* = 0.005). Furthermore, patch clamp measurements demonstrated a 3-fold increase in acetylcholine stimulated peak IKACh in atrial myocytes from GSK3β deficiency mice compared with control. Finally, western blot analysis of atrial extracts from knockout mice demonstrated increased levels of SREBP-1, GIRK1 and GIRK4 compared with control. Taken together with our prior observations, these data establish a role of increased GSK3β activity in the pathogenesis of parasympathetic dysfunction in type 1 diabetes via the regulation of IKACh and GIRK1/4 expression.

## Introduction

Diabetic Autonomic Neuropathy (DAN) is a major complication of diabetes mellitus and has been associated with a marked increase in the incidence of sudden death in diabetics [1, 2]. Sudden out of hospital cardiac death and arrhythmic death in the setting of acute MI continue to be major public health problems [3]. The incidence of sudden death was 2.9 times higher in diabetic compared with non-diabetic patients in which ventricular tachycardia (VT) was documented in at least half these patients near the time of collapse [4]. Risk factors for sudden death include clinical manifestations of parasympathetic dysfunction, such as a decreased high frequency (HF) component of heart rate variability (HRV) and increased dispersion of QT intervals [4]. More than half of the patients with diabetes for 10 years or more have an impaired response of the heart to parasympathetic stimulation characterized by a reduction in the HF component of HRV. Furthermore, studies of type 1 diabetics who die suddenly in their sleep, “dead in bed syndrome”, suggested that HRV analysis of diabetic patients who lack clinical evidence of autonomic neuropathy often demonstrate decreased parasympathetic tone [5]. Hence, decreased HRV is an important risk factor for arrhythmia and sudden death in diabetics.

Although attenuation of the parasympathetic response in the diabetic heart has been attributed to neuronal dysfunction, we have previously presented data demonstrating that decreased responsiveness of the heart to parasympathetic stimulation was also due in part to an insulin dependent decrease in expression of the molecular components of the parasympathetic response pathway in the myocardium [6, 7]. Specifically, the parasympathetic response to vagal stimulation is mediated by acetylcholine release from parasympathetic ganglia in the atrial myocardium which binds to M_2_ muscarinic receptors which activate the inward rectifying K^+^ channel (IKACh) resulting in hyperpolarization of the myocyte membrane, prolonged diastolic depolarization with a resultant slowing of the heart rate. IKACh is a heterotetrameric G-Protein Coupled Inward Rectifying K^+^ Channel (GIRK) composed of (GIRK1)_2_/(GIRK4)_2_ subunits_,_ which is activated in response to the binding of the βγ-subunit of the heterotrimeric G-protein, G_i2_, released in response to the binding of acetylcholine to the M_2_ muscarinic receptor [8]. To study the mechanism by which insulin deficiency results in parasympathetic dysfunction, we used the Akita type 1 diabetic mouse, which harbors a point mutation in the pro-insulin *ins2* (*Ins2*^Cys96Tyr^) gene which interferes with insulin processing resulting in the death of pancreatic β cells [9]. We previously demonstrated that parasympathetic dysfunction in this mouse model was due at least in part to a coordinate decrease in expression of the M_2_ muscarinic receptor, Gα_i2_, GIRK1 [6] and GIRK4 [7]. We further demonstrated that expression of GIRK1 and GIRK4 was dependent on the sterol response element binding protein (SREBP)-1, which regulates cholesterol biosynthesis and plays a role in glucose metabolism and that levels of SREBP-1 were significantly decreased in the Akita mouse heart [10–12].

Finally, we have previously presented data supporting the hypothesis that Glycogen synthase kinase (GSK)3β, a serine/threonine kinase originally identified as an enzyme that phosphorylates and down regulates glycogen synthase [10] plays a role in the pathogenesis of parasympathetic dysfunction in the diabetic heart. GSK3β is constitutively active in the basal state via phosphorylation of Tyr216 and inactivated by phosphorylation at the regulatory Ser9 residue in response to insulin stimulation of the IR (insulin receptor)/IRS (insulin receptor substrate)/PI3 kinase (PI3K)/Akt cascade [11]. Insulin deficiency has been shown to result in decreased Ser9 phosphorylation of GSK3β and a resultant increase in GSK3β activity and has been implicated in the pathogenesis of diabetic nephropathy and retinopathy [12]. We have demonstrated that GSK3β activity is increased in the heart of the Akita mouse [6, 7]. We further demonstrated that levels of SREBP-1 were significantly decreased in the Akita mouse heart due at least in part to increased GSK3β activity consistent with the work of Punga et al and Kim et al [13, 14]. Experiments with GSK3β inhibitors Li^+^ and CHIR-99021, supported the hypothesis that hyperactivity of GSK3β might play a role in the regulation of parasympathetic responsiveness via an effect on SREBP-1 regulation of GIRKs expression [7]. However, both these inhibitors lacked specificity for GSK3β [15, 16]. Here we generated an Akita mouse with cardiac specific, conditional tamoxifen inducible knockout of GSK3β to directly establish the role of GSK3β in regulation of GIRK1, GIRK4, IKACh and the parasympathetic response of the heart.

## Research design and methods

### Mice

The GSK3β^flox/flox (fl/fl)^ mouse with loxP sites flanking exon 2 of the *gsk3β* gene was generated as previously described [17]. Briefly, mice expressing an α-myosin heavy chain (α-MHC) promoter driven, tamoxifen-inducible MerCreMer (The Jackson laboratory, Bar Harbor, ME) were crossed with GSK3β^fl/fl^ to generate GSK3β^fl/fl/^Cre^+^ mice. GSK3β^fl/fl/^Cre^+^ mice were then crossed with Akita type 1 diabetic mice (C57BL/6-Ins2^Akita^/J, The Jackson laboratory) to generate Akita type 1 diabetic GSK3β^fl/fl/^Cre^+^ mice. Genotypes were confirmed by PCR for the presence of Cre and for the floxed GSK3β allele using primers shown below. As predicted: WT allele gave an 886bp band, GSK3β^fl/fl^ allele gave a 1095bp band and GSK3β knockout allele gave a 250bp band. PCR primers (Table 1) were synthesized by the Tufts University Core Facility. Genotyping of Akita mice was verified by restriction fragment length polymorphism analysis as described previously [18]. Cre expression was determined by PCR analysis. All mouse strains were on the C57BL/6J background. For GSK3β expression, male mice at 13 weeks of age were studied: WT litter mates, Akita mice or GSK3β^fl/fl^Cre^+^Akita mice treated with either 1 mg tamoxifen (I.P. injection, Sigma T5648) dissolved at a final concentration of 10 mg/mL in 10% ethanol in sterilized sunflower seed oil (Sigma S5007) or vehicle for 5 days to generate cardiomyocyte specific GSK3β knockout Akita type 1 diabetic mice or placebo treated controls. At 16 weeks of age, usually 2 weeks after the 5-day tamoxifen treatment, atria were harvested and GSK3β expression in atrial homogenates was determined by western blot analysis. All vertebrate animal-related procedures described here were approved by the Tufts Medical Center Institutional Animal Care Committee.

**Table 1:** Sequences of primers used for PCR analysis.

Genes	Forward Primer	Reverse Primer
Cre	5’-tcccgcagaacctgaagatgttc-3’	5’-ggatcatcagctacaccagagacg-3’
GSK3β	5’-ggggcaaccttaatttcatt-3’	5’-tctgggctatagctatctagtaacg-3’
Ins2	5’-tgctgatgccctggcctgct-3’	5’-tggtcccacatatgcacatg-3’

### Materials

The GIRK4 specific antibody was from Santa Cruz Biotechnology and the GIRK1 specific antibody was from Alomone Labs (Israel). SREBP-1 and β-actin antibodies were from Santa Cruz Biotechnology. GSK3β and p-GSK3β (Ser9) antibodies were from Cell Signaling Biotechnology. To monitor the progression of diabetes, measurements of urine glucose, protein and ketones were made with Keto-Diastix Reagent Strips (Bayer). Glucose was monitored using an Accu-Chek glucometer. Body weight and serum glucose levels are summarized in Table 2.

**Table 2:** Blood glucose and body weight from WT, Akita and placebo and tamoxifen treated AkitaGSK3β^fl/fl^Cre^+^ mice.

	WT (n = 10)	Akita (n = 8)	Akita/GSK3β^fl/fl^/Cre^+^ +placebo (n = 10)	Akita/GSK3β^fl/fl^/Cre^+^ +tamoxifen (n = 10)
Blood glucose (mg/dL)	133 ± 6	540 ± 20*	620 ± 35*	543 ± 17*
Body weight (g)	28.2 ± 1.5	22.8 ± 1.4*	22.7 ± 1.6*	23.8 ± 1.4*
Resting heart rate (beats/min)	560 ± 23	574 ± 14	514 ± 12	530 ± 17

All results are expressed as mean ± SEM.

*P<0.05 compared with WT control.

### Western blot analysis

Western blot analysis of atrial homogenates was carried out as described [19, 20]. Protein concentration was determined by Bradford reagent (Bio-Rad). Each sample represents tissue from atria of a single mouse.

### ECG monitoring, heart rate and HRV analysis in conscious, unrestrained mice

**Implantation of ECG transmitters:** Anesthesia was induced with inhaled 1.5% isoflurane in oxygen. An ECG signal wireless radio frequency transmitter (Data Sciences International) was implanted in mice 12 weeks of age in a subcutaneous pocket and electrodes sutured over the right pectoralis muscle and the lower left ribs. For studies of the effects of atropine and carbamylcholine on heart rate, we compared the response in WT, Akita mice or GSK3β^fl/fl^Cre^+^Akita mice 2 weeks after a 5-day treatment with either placebo or tamoxifen. **Atropine,** one week after transmitter implantation heart rates were recorded before and after challenge with atropine (0.5 mg/kg, I.P. injection) and the increase in heart rate in response to atropine determined; **carbamylcholine**, mice were injected with 1 mg/kg propranolol in order to block β-adrenergic reflex responses to carbamylcholine, once heart rate had reached a steady state, 20 minutes (S1 Fig), mice were injected with 0.2 mg/kg carbamylcholine I.P. ECGs were recorded until heart rate returned to baseline [7]. Analysis of heart rate data: Data were recorded at a sample rate of 5,000 Hz with the use of a telemetry receiver and an analog-to-digital acquisition system (Data Sciences International). The ECG signal was analyzed using custom built software: Beat-to-beat heart rate data were computed; artifacts and non-sinus rhythms were removed after manual review. All ectopic and post ectopic beats and artifacts were removed and replaced with intervals interpolated from adjacent normal beats, discarding segments where gaps accounted for over 15% of the recording segment. Average heart rates and duration of bradycardia were computed as described previously [7, 19]. **For HRV studies**: R-wave detection and beat annotation were both manually reviewed as described above. Frequency-domain analysis was performed after construction of an instantaneous RR interval time series by resampling at 10Hz. Power spectra of detrended 2-min segments were computed for frequency ranges at 0.5–1.5 Hz taken as low frequency (LF) power and 1.5–5 Hz as high frequency (HF) power and the HF fraction determined as HF/(LF+HF). HF power has been shown to result predominantly from parasympathetic modulation of heart rate with a small but significant sympathetic contribution, while LF power has been shown to result from both sympathetic and parasympathetic modulation of heart rate [21, 22]. Based on this observation, we have previously described a method for assessing parasympathetic modulation of heart rate [23]. Specifically, we inhibited the sympathetic modulation of heart rate by treating mice with the β-adrenergic blocker propranolol. Given that β-adrenergic receptor inhibition blocks the sympathetic component of HRV, leaving the parasympathetic component relatively unopposed, we computed the time course of the increase in HF fraction after the injection of propranolol. In support of this measurement of the increase in HF fraction in response to propranolol as a measure of parasympathetic responsiveness, we have previously demonstrated, that in the mouse heart HF fraction increased with a time course similar to that for the decrease in LF power and total power (TP), while HF power decreased slightly [23]. Given that we compute HF fraction as HF/(HF + LF), these findings support the conclusion that the increase in HF fraction in response to propranolol is primarily due to a decrease in LF power resulting from sympathetic blockade. Hence, the increase in the HF fraction in the response of the mouse heart to propranolol is due to parasympathetic modulation of heart rate that is not affected by sympathetic blockade. HF and LF were computed for two-minute segments at the end of the baseline and propranolol phases. In order to minimize the effects of activity of the mice, we chose segments where heart rate and frequency domain parameters were relatively stationary, verified by employing Kalman-smoothing and wavelet-based visualization in addition to FFT-based spectrograms and where noise due to mouse movement and the associated muscle activity and changes in entropy were minimal. Composite plots of HF fraction were computed from FFT power spectra over a three-minute sliding window of RR-interval data, repeated every 10 seconds and averaged to one HF fraction data point per minute per group (±SEM,). We took the value of HF fraction at 20 min after propranolol injection at which time heart rate had reached a steady state (S2 Fig). In these studies, we compared differences in HF fraction in response to propranolol in GSK3β^fl/fl^Cre^+^Akita mice before and 2 weeks after a 5-day treatment with tamoxifen. Hence each mouse served as its own control.

### Cell culture of adult mouse atrial myocytes

Dissociated atrial myocytes from mouse atria were prepared by a retrograde Langendorf perfusion method as described [6] with some modifications [7]. Cells were rod shaped with clearly defined striations.

### Cellular electrophysiology

Membrane currents were measured by the patch-clamp technique in whole-cell mode using an EPC-9 amplifier (HEKA Elektronik) as described [6]. In order to obtain and maintain good seal formation required for membrane current recording, cell contractions were suppressed with high external K^+^ and 0 external Ca^2+^, which leads to persistent membrane depolarization and inactivation of voltage-activated Na^+^ channels. Both of these conditions have been shown to have no effect on IKACh [7]. Whole cell currents were elicited at room temperature in the absence and presence of 20 μM carbamylcholine introduced by focal perfusion over 60–90 seconds. Currents were normalized to the cell capacitance determined via capacitance compensation and data presented as current density in pA/pF. Current-voltage (I-V) plots were constructed from a series of data points obtained from the carbamylcholine current responses at given voltages.

### Echocardiography

Echocardiographic studies were performed as previously [19]. We utilized a commercially available echocardiography system (Vevo 2100, VisualSonics) equipped with a MicroScan MS550D head. Anesthesia was induced with inhaled 1.5% isoflurane in oxygen and maintained with inhaled 1.0% isoflurane in oxygen. Animals were placed on a warming pad to maintain body temperature at 36.5 to 37.5°C.

### Statistics

All values are expressed as mean ± standard error of mean (SEM). Statistical differences between mean values were calculated by independent or pairwise Student's *t*-test as appropriate. Normal distribution assumptions were verified using the Shapiro-Wilk test. A *P* value < 0.05 was considered significant.

## Results

### Generation and characterization of cardiomyocyte-specific GSK3β knockout mice

**Fig 1:**
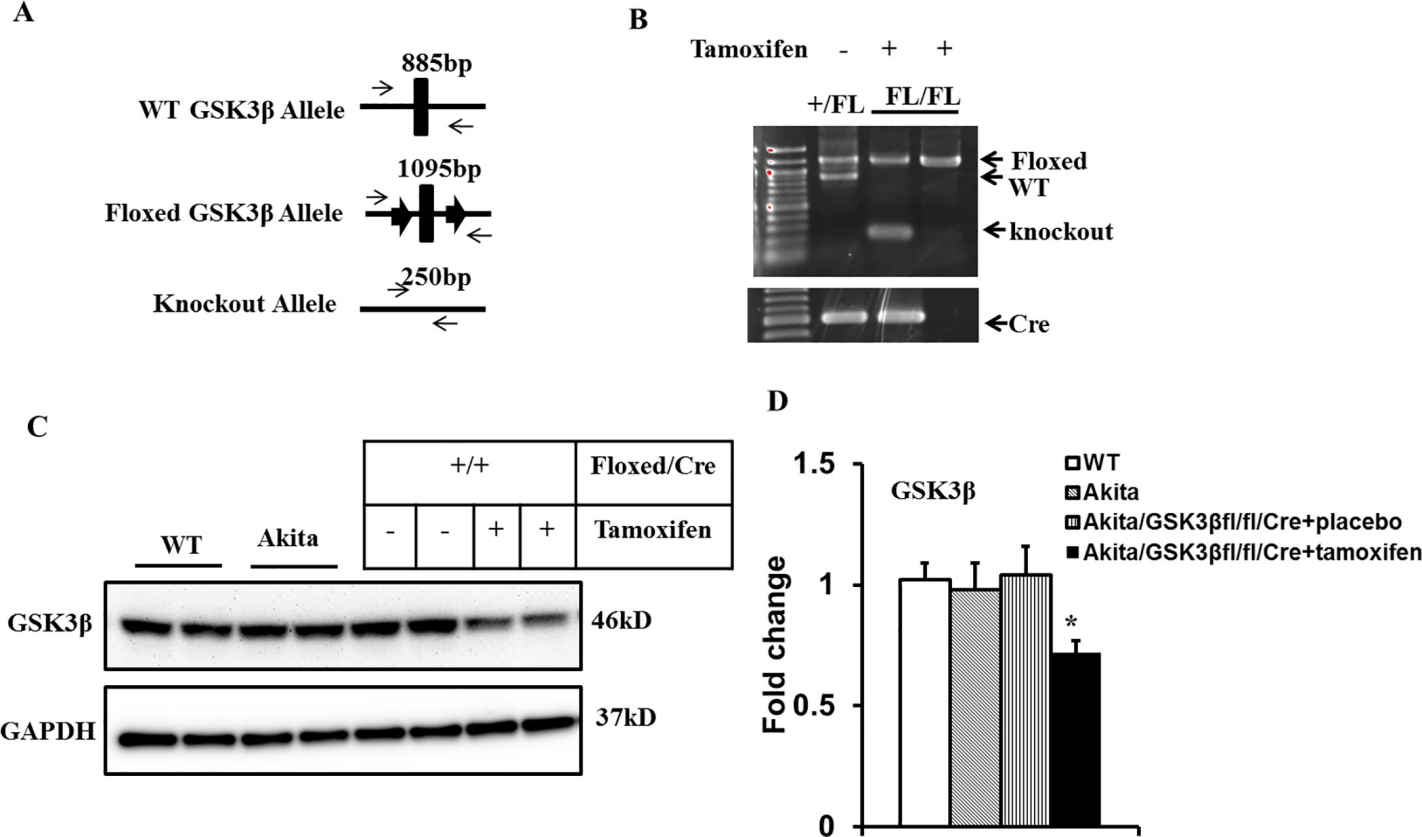
Generation of type 1 diabetic Akita mice with cardiac specific conditional knockout of GSK3β. ***A*:** Schematic outline describing the targeting strategy and predicted fragments for generating GSK3β mice with a floxed allele in exon 2 crossed with mice expressing a MerCreMer recombinase under the control of the cardiac-specific αMHC promoter. ***B*.** PCR analysis. Genomic DNA was isolated from the atria and analyzed by PCR for tamoxifen induced Cre-mediated excision of GSK3β using primers to the region flanking the floxed allele which gave bands at 885bp, 1,095bp and 250bp respectively. PCR (upper panel) demonstrated that the GSK3β floxed allele is excised only in the Cre^+^/tamoxifen injected mice. The bottom panel displays genomic detection of the Cre transgene. ***C*:** Representative immunoblots of GSK3β from atria in WT, Akita, placebo and tamoxifen treated Akita GSK3β^fl/fl^Cre^+^ mice. GAPDH was used as a loading control. ***D***: Bar graph of data in C normalized to GAPDH. Results are reported as mean ± SEM, *P<0.05, N = 5 for each.

To evaluate the role of GSK3β in the regulation of parasympathetic dysfunction in atrial cardiomyocytes from Akita type 1 diabetic mice, we generated an Akita mouse with a conditional, cardiac specific deletion of GSK3β. Initially, we crossed GSK3β^fl/fl^ mice with mice expressing tamoxifen-inducible MerCreMer, in which the expression of the Cre recombinase was under the control of the αMHC-promoter to generate GSK3β^fl/fl^Cre^+^ mice. The progeny were then crossed with Akita mice to obtain tamoxifen inducible cardiomyocyte-specific GSK3β knockout Akita type 1 diabetic mice. PCR analysis of genomic DNA isolated from the atria demonstrated the predicted bands in WT, GSK3β^fl/fl^Cre^+^Akita mice and tamoxifen treated GSK3β^fl/fl^Cre^+^Akita mice (Fig 1A and 1B, *Methods*). Mice with the Akita mutation demonstrated glucose levels > 500mg/dL. Western blot analysis demonstrated no significant difference in GSK3β protein between WT, Akita, and placebo treated GSK3β^fl/fl^Cre^+^Akita mice, while tamoxifen treatment of GSK3β^fl/fl^Cre^+^Akita mice resulted in a ≈30% reduction of GSK3β protein in the atria compared with placebo treated controls (0.72 ± 0.05 vs. 1.00 ± 0.12, n = 5, p<0.05) (Fig 1C and 1D). Comparison of WT, Akita or placebo and/or tamoxifen treated Akita GSK3β^fl/fl^Cre^+^ mice demonstrated that neither the presence of the floxed allele nor cardiac specific deletion of GSK3β had an effect on the level of hyperglycemia and the decrease in body weight of Akita type 1 diabetic mice (Table 2).

Echocardiographic analysis also demonstrated that cardiac specific deletion of GSK3β at the age of 4 months had no effect on left ventricular end diastolic dimension, left ventricular end systolic dimension, fractional shortening, ejection fraction, or resting heart rate (S1 Table).

### Decreased expression of GSK3β in atria of the Akita mouse reverses the parasympathetic dysfunction associated with type 1 diabetes

**Fig 2:**
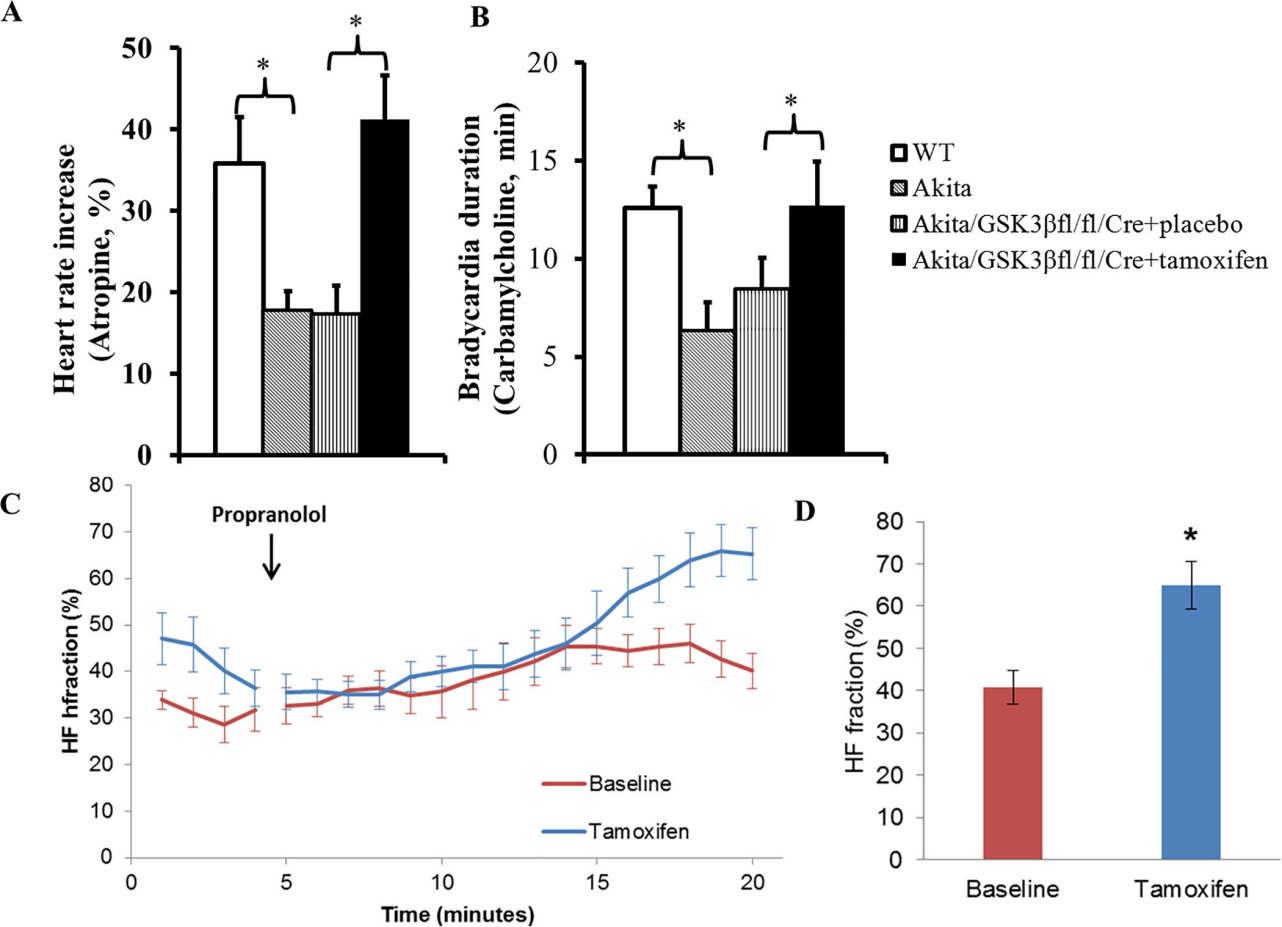
Effect of GSK3β deficiency on parasympathetic response in Akita mice. ECGs were monitored in WT, Akita and GSK3β^fl/fl^Cre^+^Akita mice, the latter studied 2 weeks after a 5-day treatment with either placebo or tamoxifen. Mice received an intraperitoneal injection with either **A:** 0.5 mg/kg atropine and monitoring continued for 30 min and the % increase in heart rate determined. The maximal heart rate response to atropine occurs 3–4 min after injection; or ***B*:** 1mg/kg propranolol followed 20 min later by 0.2 mg/kg carbamylcholine and monitoring continued until recovery of heart rate to baseline. The duration of bradycardia was determined as the length of the plateau phase of the bradycardic response [1]. Results are reported as mean ± SEM. Effect of GSK3β deficiency on HF fraction **C.** Group wise comparison of the time course of averaged (±SEM) composite plots of HF fraction in GSK3β^fl/fl/^Cre^+^Akita mice in response to injection of 1mg/kg propranolol in the same mice studied before (baseline) and 2 weeks after 5 days of treatment with tamoxifen. Here each mouse serves as its own control. Measurements were made over segments during which heart rates were stable (See Methods). **D**. Quantitation of HF fraction. Statistical comparisons were made by Student *t* test throughout the figure. *P<0.05, (n values in the text).

Given that resting heart rate reflects the balance between the response of the heart to sympathetic and parasympathetic stimulation, the increase in heart rate in response to muscarinic blockade by atropine reflects the level of parasympathetic modulation of heart rate. Thus, the increase in heart rate in response to atropine and the decrease in heart rate in response to the muscarinic agonist carbamylcholine are both measures of parasympathetic responsiveness. **Atropine response:** To determine the effect of cardiac specific deletion of GSK3β on the parasympathetic response, we compared the heart rate response to atropine in WT, Akita and in GSK3β^fl/fl/^Cre^+^Akita mice 2 weeks after a 5-day treatment with either placebo and/or tamoxifen. In WT mice heart rate increased from a resting rate of 560 ± 23 beats/min to 760 ± 25 beats/min after atropine, or 35.8 ± 5.7% (n = 8, P<0.05). In Akita mice heart rate increased from a resting rate of 574 ± 14 beats/min to 671 ± 21 beats/min after atropine, or 17.9 ± 2.3% (n = 8, P<0.05). Finally, the heart rate response to atropine in placebo treated GSK3β^fl/fl/^Cre^+^Akita mice increased from 514 ± 12 beats/min prior to atropine to 610 ± 18 beats/min or 17.3 ± 3.5% (n = 8) which was not significantly different from that seen in Akita mice. However, in tamoxifen treated mice the atropine response increased from 530 ± 17 beats/min to 713 ± 19 beats/min or 41.2 ± 5.4% (n = 8, *P*<0.05; Fig 2A). Importantly, note that the resting heart rate was not significantly different in all 4 sets of mice, Table 2 and above. **Carbamylcholine response:** For the study of the effect of GSK3β deficiency in Akita mice on the heart rate response to the acetylcholine analog carbamylcholine, mice were pretreated with propranolol to block the β-adrenergic reflex response to carbamylcholine. Propranolol blockade at this concentration was shown to reach a steady state heat rate in mice within 4–8 minutes which persisted for up to 40 min (S1 Fig) [24]. Animals were subsequently challenged with carbamylcholine, and heart rate determined [6, 7]. The duration of bradycardia after carbamylcholine administration, defined as the time over which the maximum decrease in heart rate was stable before the initiation of recovery, was 8.43 ± 1.60 min in placebo treated GSK3β^fl/fl/^Cre^+^ Akita mice and 12.71 ± 2.26 min in tamoxifen treated mice (n = 8, P<0.05, Fig 2B). Importantly the duration of bradycardia in WT mice was 12.60 ± 1.07 min (n = 8), and 6.28 ± 1.51 min (n = 8) in Akita mice. The finding that decreased expression of GSK3β in the atrium markedly increased the response of the heart to parasympathetic stimulation to a level similar to that in WT mice supported the conclusion that parasympathetic dysfunction in Akita mice was due at least in part to increased GSK3β activity in the atria.

### Decreased GSK3β expression in the Akita mouse heart increases parasympathetic response as measured by HRV

We had previously determined that HRV was significantly decreased in the Akita mouse heart [7]. To determine the effect of attenuation of GSK3β on HRV, we analyzed HRV before and 2 weeks after a 5-day treatment with tamoxifen. This approach permitted each mouse to serve as its own control. Given that β-adrenergic receptor inhibition blocks the sympathetic component of HRV, leaving the parasympathetic component relatively unopposed, and HF power has been shown to result predominantly from parasympathetic modulation of heart rate with a small but significant sympathetic contribution, while LF power has been shown to result from both sympathetic and parasympathetic modulation of heart rate [21, 22] we had previously demonstrated that the increase in HF fraction, HF/(HF+LF) of HRV was due primarily to a decrease in LF and hence served as a measure of parasympathetic response (See methods). A plot of HF fraction over time demonstrated a continuously increasing response reaching a plateau at 15 minutes in GSK3β^fl/fl^Cre^+^Akita mice studied prior to tamoxifen treatment and at 20 minutes in the same mice studied 2 weeks after 5 days of tamoxifen treatment. The mean HF fraction was higher in tamoxifen treated mice, 65.04 ± 5.64%, compared to 40.78 ± 3.86% (n = 10, P<0.05, Fig 2C and 2D) in the same mice studied prior to tamoxifen. Interestingly, these values were similar to those previously reported in WT and Akita mice respectively [23] consistent with the conclusion that attenuation of GSK3β resulted in reversal of the parasympathetic dysfunction in the Akita mouse.

### Decreased expression of GSK3β in the atrium increased IKACh in atrial cardiomyocytes from Akita diabetic mice

**Fig 3:**
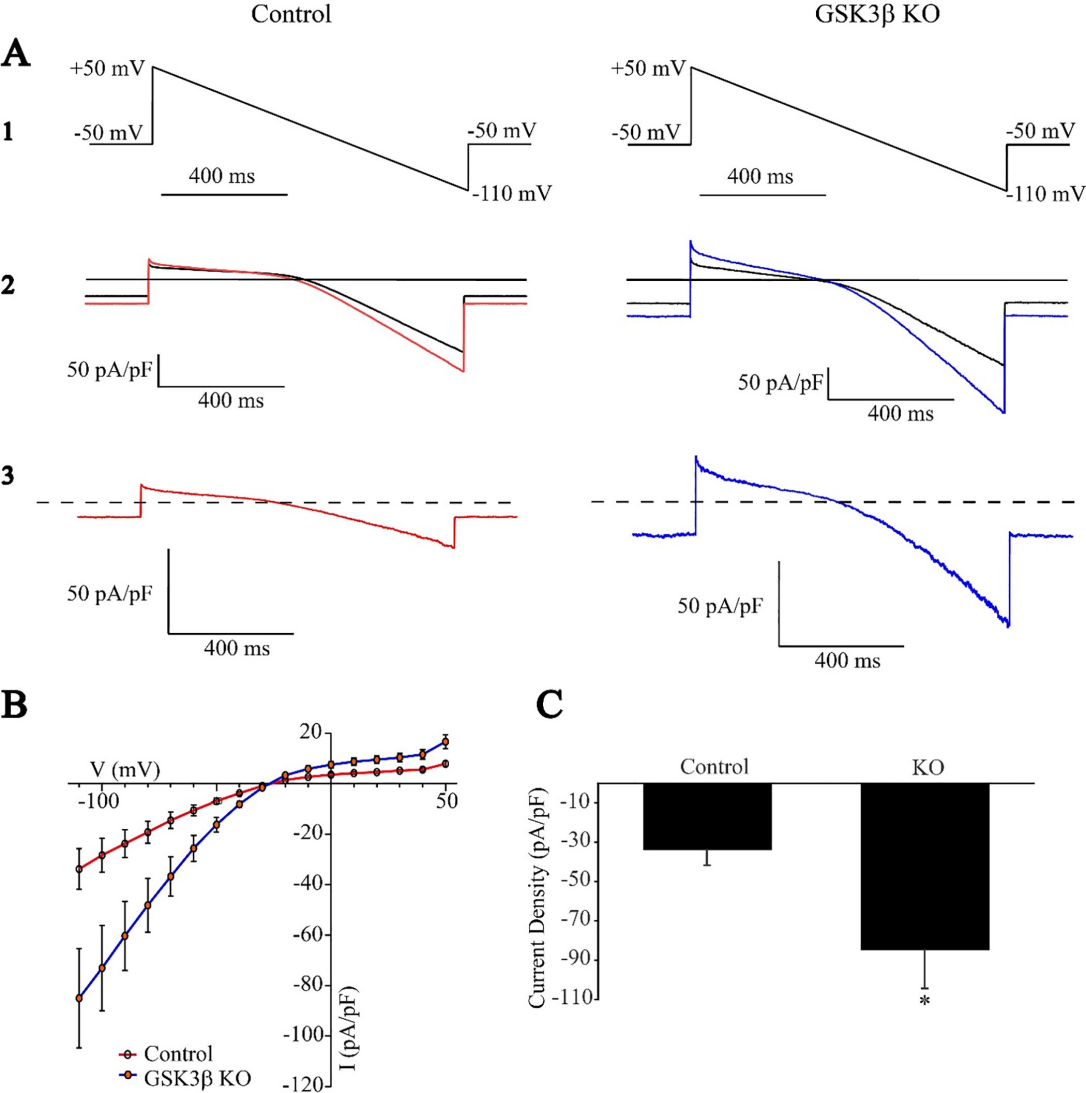
IKACh is increased in atrial myocytes from tamoxifen treated GSK3β^fl/fl^ Cre^+^Akita (KO) mice compared with placebo treated GSK3β^fl/fl^ Cre^+^ Akita mice (Control). IKAch was determined as described in Methods, and I-V plots were constructed. ***A*:** I-V relationship of the carbamylcholine-induced whole-cell currents elicited from a 1-second voltage ramp with a continuously changing voltage from +50 to -110 mV **(1)**; Current from a typical atrial myocyte with and without 20 μmol/L carbamylcholine **(2)**; Current generated by subtracting the trace obtained prior to and after the addition of carbamylcholine **(3)**. ***B*:** I-V plots constructed from a series of data points as in *A*3. Data are the mean ±SEM of 9 recordings from cells from 4 vehicle treated GSK3β^fl/fl^/Cre^+^Akita mice and 10 recordings from 4 tamoxifen treated GSK3β^fl/fl^/Cre^+^Akita mice. ***C*:** Quantitation of peak inward currents from **B**, *P<0.05 compared to control.

To determine whether GSK3β played a role in the regulation of IKACh, and whether the increased parasympathetic response of the heart in the GSK3β knockout mouse was associated with an increase in IKACh, we compared IKACh in atrial myocytes from GSK3β^fl/fl^Cre^+^Akita mice which had been treated either with vehicle and/or tamoxifen. Atrial myocytes from these mice were prepared as described [1]. Cells were rod-shaped and demonstrated clearly defined striations and spontaneous contractions with stable resting membrane potentials. Cell size determined as cell capacitance was not significantly different between groups (71.5 ± 4.3 pF, n = 12 in control vs 66.3 ± 5.3 pF, n = 20 in KO, p = 0.45). The current-voltage (I-V) relationships derived from membrane currents in these cells demonstrated that myocytes from atria of GSK3β^fl/fl^Cre^+^Akita mice treated with tamoxifen exhibited a marked increase in carbamylcholine stimulated IKACh with a peak inward current of -221.1 ± 29.5 pA/pF (n = 10) compared to -134.9 ± 12.6 pA/pF (n = 9, *P*<0.05) in atrial myocytes from vehicle treated GSK3β^fl/fl^Cre^+^Akita mice (Fig 3A, 3B and 3C).

### Mechanism of increased parasympathetic response in GSK3β deficient Akita mice

**Fig 4:**
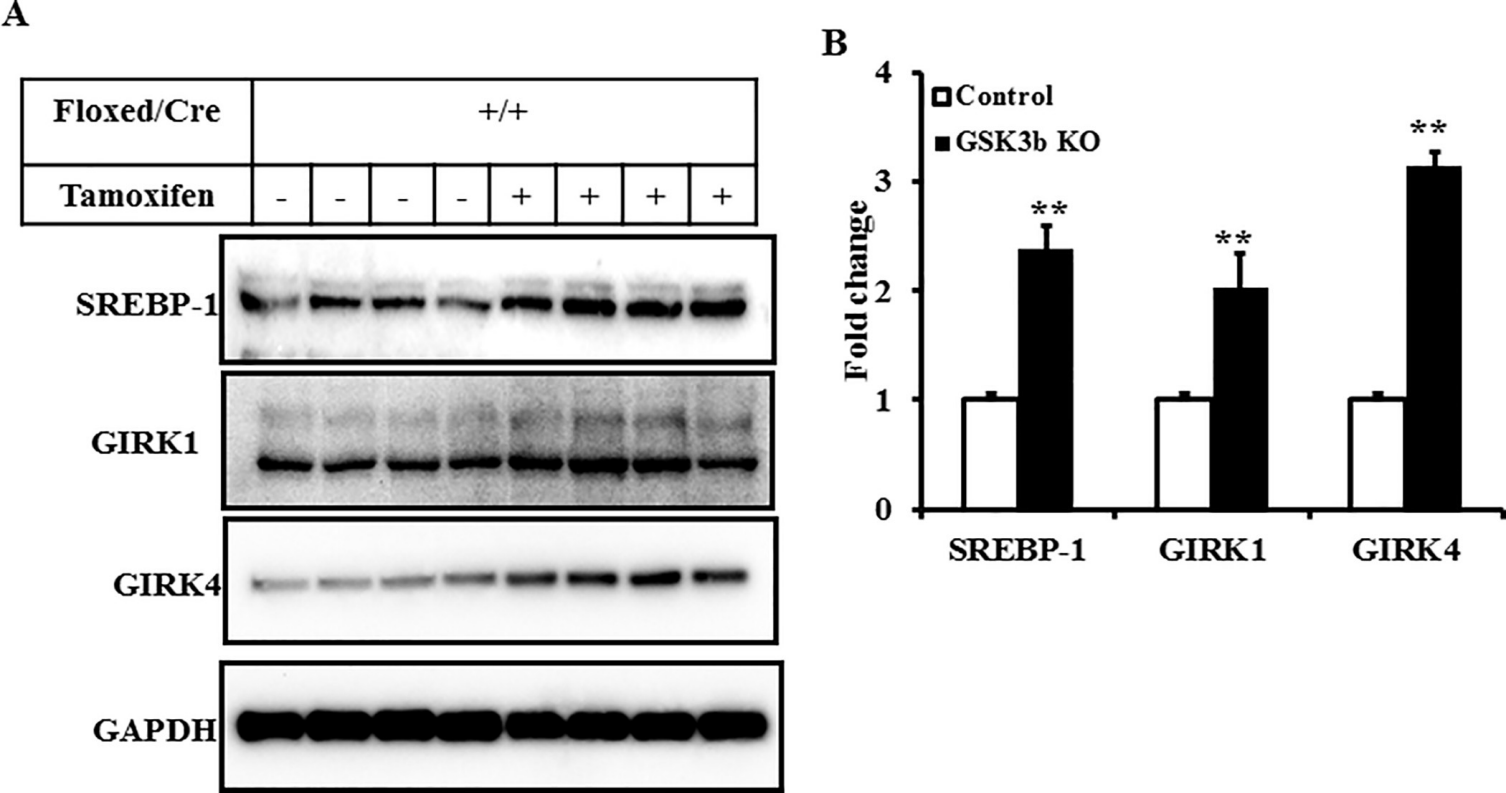
Decreased GSK3β expression in atria of Akita mice resulted in increased levels of SREBP-1 and GIRK1/4 *A*. Levels of SREBP-1 and GIRK1/4 in atria from placebo and tamoxifen treated GSK3β^fl/fl/^Cre^+^ Akita mice determined by Western blot analysis of atrial extracts of 6 mice in each group. ***B*:** Densitometry analysis of SREBP-1 and GIRK1/4 from *A*. Data were normalized to the expression of GAPDH. **P<0.01 compared to control Akita mice.

To determine the effect of knockout of GSK3β in the Akita mouse on the expression of proteins that mediate parasympathetic responsiveness in the heart and IKACh activity in atrial myocytes, we compared levels of the molecular components of the parasympathetic response pathway, including GIRK1 and GIRK4 and the transcription factor SREBP-1 in atria of placebo and tamoxifen treated GSK3β^fl/fl^Cre^+^Akita mice [6, 7, 19]. We had previously determined that levels of SREBP-1 and GIRK1/4 in atria of Akita mice were decreased compared to WT in parallel with decreased parasympathetic responsiveness and decreased IKACh in atrial myocytes form these mice and that adenoviral overexpression of nSREBP-1 in atrial myocytes from Akita mouse hearts increased IKACh consistent with the conclusion that expression of GIRK1/GIRK4 was under the control of SREBP-1 [6, 7]. Decreased expression of GSK3β in atria of Akita mice resulted in an increase in the levels of SREBP-1, GIRK1 and GIRK4 by 2.39 ± 0.21 (n = 6, *P*<0.01), 2.03 ± 0.32 (n = 6, *P*<0.01) and 3.14 ± 0.14 (n = 6, *P*<0.01) fold, respectively compared to control (Fig 4). Thus, decreased expression of GSK3β in atria of Akita reversed the effects of insulin deficiency on parasympathetic dysfunction, IKACh and gene expression.

## Discussion

The onset of Cardiac Autonomic Neuropathy has been attributed to vagal nerve dysfunction. However, a number of animal models for diabetes including an alloxan treated rabbit model demonstrating impaired baroreceptor mediated bradycardia, a streptozotocin treated rat model and our more recent studies in the Akita type 1 diabetic mouse support the conclusion that parasympathetic dysfunction might also be associated at least in part with a decrease in the ability of the end organ to respond to parasympathetic stimulation [6, 7, 25, 26]. Specifically, studies in our laboratory demonstrated that parasympathetic dysfunction is at least in part due to decreased expression of proteins which mediate the response of the heart to parasympathetic stimulation including the M_2_ muscarinic receptors, the G-protein Gα_i2_ and the structural components of the IKACh ion channel, GIRK1 and GIRK4 in atrial myocytes [6]. We further demonstrated that the transcription factor SREBP-1 which regulates GIRK1 and GIRK4 at the level of transcription is also decreased in the Akita mouse.

Insulin deficiency has been associated with increased activity of GSK3β due to the role of insulin in the regulation of GSK3β activity via the PI3kinase mediated phosphorylation of GSK3β at Ser9. Hyperactivity of GSK3β has been implicated in secondary effects of diabetes in the kidney and retina [11]. However, the mechanism by which increased GSK3β activity in the heart might regulate cardiac functions has not been well described. Here we demonstrate that cardiac specific partial conditional KO of GSK3β in the atrium of Akita mice results in a marked reversal of the parasympathetic dysfunction observed in the Akita mouse as measured by an increase in the HF fraction of HRV and of the magnitude of the increase in heart rate in response to parasympathetic blockade by atropine and the decrease in heart rate in response to carbamylcholine treatment. We further demonstrate that this increase in parasympathetic responsiveness in the Akita GSK3β KO mouse is associated with an increase in the magnitude of carbamylcholine stimulated IKACh. The finding that partial KO of GSK3β in the Akita mouse results in increased levels of GIRK1 and GIRK4 expression strongly supports the conclusion that hyperactivity of GSK3β in the diabetic heart results in parasympathetic dysfunction via an effect on the level of GIRK proteins. Furthermore, given our prior studies implicating SREBP-1 in the regulation of GIRK1 and GIRK4 expression at the level of transcription, the finding that KO of GSK3β in the Akita mouse was associated with increased SREBP-1 further supported the hypothesis that hyperactivity of GSK3β resulted in decreased levels of GIRK proteins via an effect on SREBP-1. The increase in SREBP-1 expression in response to decreased GSK3β activity is consistent with the findings of Punga et al and Kim et al who demonstrated that GSK3β mediated SREBP-1 turnover via ubiquitination and proteosomal degradation [13, 14]. These data support the conclusion that hyperactivity of GSK3β is at least in part responsible for the decreased HRV and atropine response in the type 1 diabetic Akita mouse due to decreased expression of the GIRK1 and GIRK4 subunits of IKACh in response to a decrease in SREBP-1.

Significant data rule out the possibility that tamoxifen alone might account for the observed increase in parasympathetic responsiveness in the Akita mouse. Specifically, the 5 day tamoxifen treatment is always followed by at least a 2-week period before studies are initiated to allow for both wash out of the drug and excision of the floxed allele [27, 28]. Furthermore, in a study of the effect of KO of N-Cadherin on ECG changes in heart rate and ventricular arrhythmia, tamoxifen treatment of Cre^+^ mice with the floxed N-Cadherin allele had no effect on ECG or arrhythmogenesis [29]. Most importantly, data in S1 Table demonstrated that tamoxifen treatment of GSK3β^fl/fl^/Cre^+^Akita mice had no significant effect on cardiac function, chamber size and wall thickness.

These findings were consistent with our prior findings that Li^+^ and small molecule inhibitors of GSK3β appeared to reverse parasympathetic dysfunction in the Akita mouse [7]. However, these prior studies were complicated by a lack of specificity of these agents. Importantly, Li^+^ has been shown not only act as a GSK3β inhibitor, but also to effect membrane potential, Na^+^ currents and Mg^2+^ dependent processes, while small molecule inhibitors of GSK3β block enzyme activity by impeding access of ATP to its binding site for inhibition of GSK3β and GSK3α [15, 16]

The generation of a type 1 diabetic Akita mouse with conditional cardiac specific KO of GSK3β has permitted us to directly establish the role of hyperactivity of GSK3β in the pathogenesis of parasympathetic dysfunction in this mouse model. Interestingly, knockout efficiency of GSK3β was only 30% in the Akita atrium, while knockout efficiency was 90% in the ventricle (S2 Fig). This suggests that although the αMHC promoter is specific for both atrium and ventricle, its ability to drive Cre expression might be significantly higher in the ventricle. More importantly, the finding that the decrease in the level of GSK3β in the atrium reported here is sufficient to reverse the effects of hypoinsulinemia on parasympathetic dysfunction and expression of GIRK1/GIRK4 strongly supports the conclusion that levels of this enzyme are limiting in the atrium and directly establish a role for hyperactivity of GSK3β in the pathogenesis of autonomic dysfunction in this mouse model for type 1 diabetes.

It has been suggested that L-type Ca^2+^ channel activity might be decreased in the Akita mouse heart [30], which might also account for autonomic dysfunction. However, we have previously demonstrated that at the ages studied, resting L-type Ca^2+^ channel activity is not significantly different in ventricular myocytes from Akita and WT mice at the ages studied [31]. We previously developed a unique approach for the study of parasympathetic modulation of the heart rate by computing the HF fraction after the inhibition of the sympathetic contribution to the power spectrum in response to a propranolol injection. By using this approach, differences in the HF fraction after sympathetic blockade in WT and Akita mice would be due primarily to differences in the parasympathetic contribution to HF power [23]. The finding that attenuation of GSK3β activity results in an increased heart rate response to atropine, and an increase in the bradycardia response to carbamylcholine taken together with the increased HF fraction of HRV in response to propranolol in these mice further strongly support the conclusion that GSK3β plays a critical role in regulating the autonomic balance in the Akita mouse.

GSK3β has been implicated in cardiomyocyte proliferation during embryonic development of the heart, in post myocardial infarction remodeling and in cardiac hypertrophy [32]. GSK3β has also been implicated in the development of insulin resistance and in death of pancreatic beta cells in models of type 2 diabetes. Inhibition of GSK3β via the over-expression of metallothionine has been shown to attenuate the development of ventricular remodeling in the development of diabetic cardiomyopathy [33]. Thus, significant data implicate GSK3β in the pathogenesis of cardiovascular disease. As noted, the molecular pathways responsible for these effects of GSK3β are not well described. Taken together with prior data demonstrating that SREBP-1 regulates the expression of the structural subunits of the IKACh channel and hence IKACh activity [6, 7], the finding that cardiac specific attenuation of GSK3β activity in the atrium of the Akita mouse results in decreased levels of SREBP-1 and increases in both IKACh activity and expression of GIRK1/GIRK4 further supports the relationship between GSK3β and SREBP-1 and its role in the regulation of parasympathetic responsiveness in the heart. These findings support a new mechanism for the effects of hyperactivity of GSK3β in the pathogenesis of parasympathetic dysfunction in type 1 diabetes. These data further establish GSK3β and the GSK3β signaling pathway as potential therapeutic targets in the treatment and prevention of autonomic dysfunction in diabetic patients and establish the GSK3β knockout mouse as an important tool in the study of the pathogenesis and treatment of diabetic autonomic neuropathy.

## Supporting information

S1 Fig Time course of heart rate response to propranolol. One week after implantation of ECG transmitters, heart rates were recorded before and after challenge with 1 mg/kg propranolol with the use of a telemetry receiver and an analog-to-digital acquisition system (Data Sciences International). Heart rates were analyzed as the mean of the response of 4 mice using DSI analysis software (n = 4).
(TIF)

S2 Fig GSK3β expression in the ventricle from WT, Akita, and GSK3β^fl/fl^ Cre^+^ Akita mice treated with Placebo or tamoxifen. **A:** Representative immunoblots of GSK3β from ventricles of WT, Akita; placebo and tamoxifen treated GSK3β^fl/fl^Cre^+^Akita mice. GAPDH was used as a loading control. **B:** Bar graph of data in **A** normalized to GAPDH. Results are reported as mean ± SEM, *P<0.05, n = 3 for each.
(TIF)

S1 Table Echocardiographic analysis of LV structure and function of Akita/GSK3β^fl/fl^/Cre^+^ mice after placebo or tamoxifen treatment. (DOCX)
