# Supplementary figures and images for: Targeted disruption of glycogen synthase kinase-3β in cardiomyocytes attenuates cardiac parasympathetic dysfunction in type 1 diabetic Akita mice
Source: PLoS One. 2019 Apr 12;14(4):e0215213. doi: 10.1371/journal.pone.0215213 (PMC6461277; doi:10.1371/journal.pone.0215213)

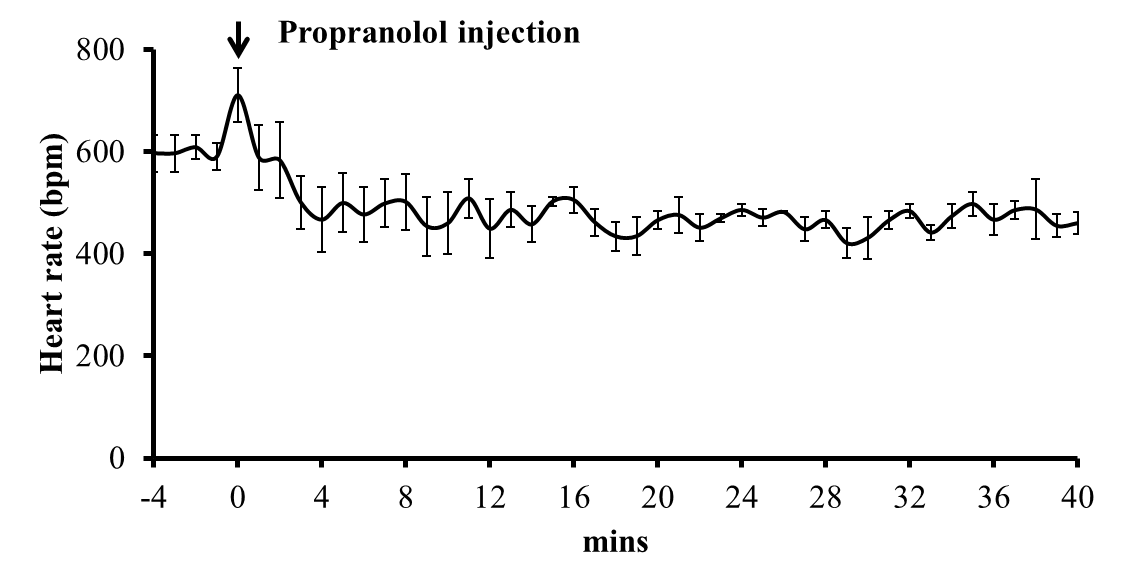

Supplement: S1 Fig — One week after implantation of ECG transmitters, heart rates were recorded before and after challenge with 1 mg/kg propranolol with the use of a telemetry receiver and an analog-to-digital acquisition system (Data Sciences International). Heart rates were analyzed as the mean of the response of 4 mice using DSI analysis software (n = 4). (TIF) [file pone.0215213.s001.tif]

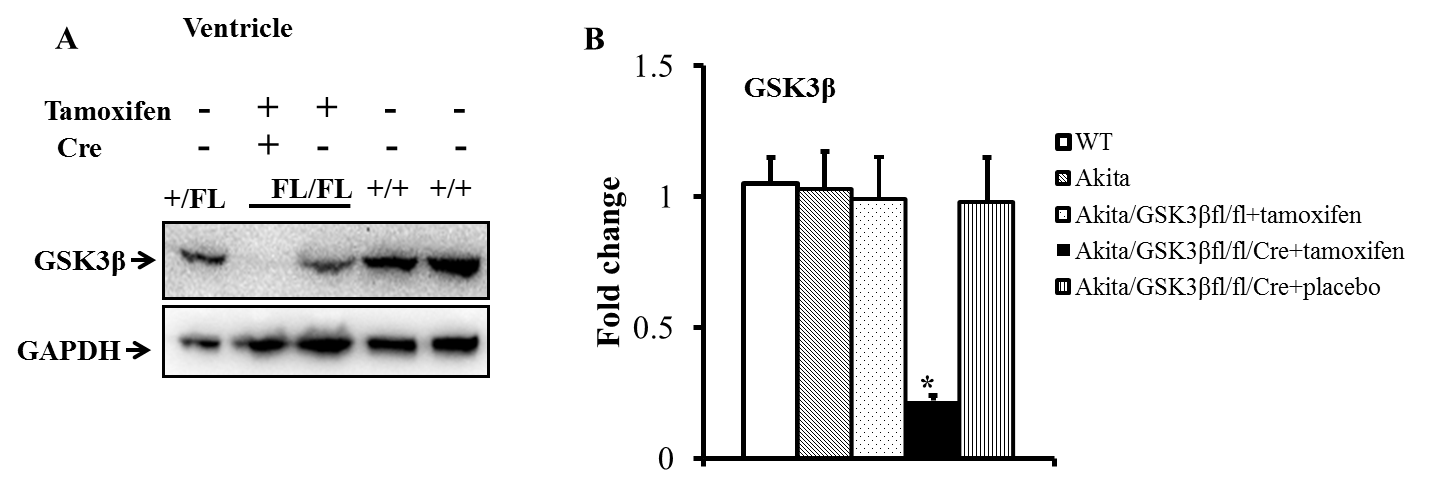

Supplement: S2 Fig — A: Representative immunoblots of GSK3β from ventricles of WT, Akita; placebo and tamoxifen treated GSK3βfl/flCre+Akita mice. GAPDH was used as a loading control. B: Bar graph of data in A normalized to GAPDH. Results are reported as mean ± SEM, *P<0.05, n = 3 for each. (TIF) [file pone.0215213.s002.tif]
