# Supplementary material for: Targeted disruption of glycogen synthase kinase-3β in cardiomyocytes attenuates cardiac parasympathetic dysfunction in type 1 diabetic Akita mice
Source: PLoS One. 2019 Apr 12;14(4):e0215213. doi: 10.1371/journal.pone.0215213 (PMC6461277; doi:10.1371/journal.pone.0215213)
Supplement: S1 Table — (DOCX) [file pone.0215213.s003.docx]

**S1 Table Echocardiographic analysis of LV structure and function of Akita/GSK3β^fl/fl^/Cre^+^ mice** **after placebo or tamoxifen treatment**

|  | Akita/GSK3βfl/fl/Cre  +placebo (n=6) | | Akita/GSK3βfl/fl/Cre  +tamoxifen (n=6) | *P* value |
| --- | --- | --- | --- | --- |
| EDD (mm) | | 3.95 ± 0.09 | 3.89 ± 0.06 | 0.135 |
| ESD (mm) | | 2.73 ± 0.08 | 2.39 ± 0.08 | 0.690 |
| Post wall (mm) | | 1.24 ± 0.05 | 1.18 ± 0.04 | 0.725 |
| Ant wall (mm) | | 1.03 ± 0.05 | 1.07 ± 0.05 | 0.642 |
| HR (beats/min) | | 398 ± 20 | 406±14 | 0.350 |
| FS (%) | | 33.01 ± 2.09 | 38.4 ± 2.11 | 0.437 |
| EF (%) | | 59.14 ± 2.98 | 69.36 ± 2.57 | 0.264 |

LV end-diastolic diameter (EDD) and end-systolic diameter (ESD) were averaged over five cardiac cycles. Fractional shortening (FS) and ejection fraction (EF) were calculated using the standard equation FS (%) = (EDD – ESD)/EDD X 100. Ejection fraction (EF) using the standard equation: EF (%) = (LVEDV - LVESV) X 100/ LVEDV; LVEDV (LV end diastolic volume) = (7 X LVEDd3)/(2.4 + LVEDd); LVESV (LV end systolic volume) = (7 X LVESd3)/(2.4 + LVESd). Post wall, posterior wall thickness; Ant wall, anterior wall thickness; HR, heart rate; FS, fractional shortening; EF, ejection fraction.
